# Management of Metastatic Spinal Cord Compression in Secondary Care: A Practice Reflection from Medway Maritime Hospital, Kent, UK

**DOI:** 10.3390/jpm11020110

**Published:** 2021-02-09

**Authors:** Sidrah Shah, Mikolaj Kutka, Kathryn Lees, Charlotte Abson, Maher Hadaki, Deirdre Cooke, Cherie Neill, Matin Sheriff, Afroditi Karathanasi, Stergios Boussios

**Affiliations:** 1 Department of Medical Oncology, Medway NHS Foundation Trust, Windmill Road, Gillingham ME7 5NY, Kent, UK; sidrah.shah@nhs.net (S.S.); mikolaj.kutka@nhs.net (M.K.); deirdrecooke@nhs.net (D.C.); cherie.neill@nhs.net (C.N.); a.karathanasi@nhs.net (A.K.); 2 Kent Oncology Centre, Maidstone and Tunbridge Wells NHS Trust, Hermitage Lane, Maidstone ME16 9QQ, Kent, UK; kathrynlees@nhs.net (K.L.); cabson@nhs.net (C.A.); mhadaki@nhs.net (M.H.); 3 Department of Urology, Medway NHS Foundation Trust, Windmill Road, Gillingham ME7 5NY, Kent, UK; matin.sheriff@nhs.net; 4 King’s College London, Faculty of Life Sciences & Medicine, School of Cancer & Pharmaceutical Sciences, London SE1 9RT, UK; 5 AELIA Organization, 9th Km Thessaloniki-Thermi, 57001 Thessaloniki, Greece

**Keywords:** metastatic spinal cord compression, corticosteroids, decompressive surgery, palliative radiotherapy, pathway

## Abstract

Introduction: Malignant spinal cord compression (MSCC) is one of the most devastating complications of cancer. This event requires rapid decision-making on the part of several specialists, given the risk of permanent spinal cord injury or death. The goals of treatment in spinal metastases are pain control and improvement of neurological function. There can be challenges in delivering prompt diagnosis and treatment in a secondary care setting. We have reflected on the experience of managing MSCC in a district general setting. Aim: Our retrospective audit identified 53 patients with suspected MSCC who entered the relevant pathway from April 2017 to March 2018 at Medway, United Kingdom (UK). Our audit standards were set out by Medway Maritime Hospital and Maidstone and Tunbridge Wells NHS Trust MSCC working group members, using a combination of published evidence and best practice. Results: The patients with suspected MSCC were 53 and 29 of them (54.7%) had confirmed MSCC. The most common malignancies within the confirmed MSCC were lung (11 patients, 37.9%), breast (5 patients 17.2%), and renal (3 patients, 10.3%), followed by prostate, myeloma and carcinoma of unknown primary (2 patients (6.9%) each), as well as pancreatic, colorectal, lymphoma and, bladder (1 patient (3.4%) each). A magnetic resonance imaging (MRI) scan was performed in 48 patients (90.5%); the majority (31 patients, 64.6%) underwent the MRI within the first 24 h, whereas 3 patients had the investigation between 24 and 72 h from the admission. Among the 29 patients with confirmed MSCC, 6 (20.6%) were treated with surgical decompression, while 20 (69%) received radiotherapy (RT) and 3 (10.3%) best supportive care, respectively. Median time to surgery was 5 days (ranged between 2 and 8 days), whereas for RT 44.4 h (ranged between 24 and 72 h). Finally, all 3 patients that decided on symptom control were referred to a palliative care team within the first 24 h following the MRI scan. Conclusions: MSCC is frequently presented outside tertiary care. This may cause subsequent delays in investigation, diagnosis, and treatment, which can be improved by following a fast track referral pathway.

## 1. Introduction

Metastatic spinal cord compression (MSCC) remains a challenging oncological emergency and requires effective multidisciplinary management for optimal effects on patients’ morbidity and quality of life [1]. Diagnosis and prompt treatment can be difficult due to patient, clinician, and institutional factors. MSCC can present with a range of symptoms from minor sensory, motor, or autonomic disturbances to severe pain and complete paraplegia.

MSCC is a complication of cancer that occurs in 5–10% of patients and can particularly complicate the final stages of their disease [2]. However, it could also be the presenting symptom of a malignancy; in a retrospective cohort study, 21% of patients with MSCC had no diagnosis of cancer within the last year [3]. The exact incidence of cases in England and Wales is not clarified, as cases are not systematically recorded, but the NICE guideline approximates cases to be 4000 in England and Wales annually [4]. The median age of diagnosis is 65 years and 60% of cases are found in lung, breast, and prostate cancers [5].

Studies have established that the mobility of patients at the time of diagnosis is a significant prognostic factor of mobility after MSCC treatment [6]. Therefore, to avoid serious neurological implications of MSCC, it is crucial that diagnosis is made as early as possible. There are clear recommendations that patients should have rapid access to magnetic resonance imaging (MRI), appropriate surgery, and radiotherapy (RT), under an MSCC coordinator. Once the diagnosis of MSCC is suspected, patients with neurological deficits should receive prompt administration of dexamethasone. Local management strategies generally include palliative RT, or surgical posterior decompression with or without instrumentation or total en bloc spondylectomy [7]. This audit study aimed to determine whether the current practice and management of MSCC at Medway, United Kingdom (UK), reflected the National Institute for Health and Care Excellence (*NICE*) guidelines.

## 2. Materials and Methods

Our retrospective study identified 53 patients with suspected MSCC in the electronic database of Medway Maritime Hospital in UK, who entered the relevant pathway from April 2017 to March 2018. Details collected were: clinical presentation, referral timing, cancer type, and whether surgery and/or RT were carried out as a definitive treatment for MSCC. Moreover, time from admission to MRI scan was also recorded. Our audit standards were set out by our MSCC working group members using a combination of published evidence and best practice [7,8].

Data was collected and analysed in Microsoft Excel and we also compared the results of our service with NICE guidelines on management of patients with MSCC.

Multiple aspects of the NICE guidelines were compared with our clinical practice in order to identify limitations and provide recommendations for improvement. The aims of our study were to:

(1) Assess if the diagnosis of MSCC was recognized by medical physicians within a timely manner,
(2) Assess whether MRI was performed within 24 h of suspicion of MSCC,
(3) Explore the treatment options provided to patients,
(4) Evaluate the performance of the multidisciplinary team in providing treatment within 24 h of diagnosis of MSCC.

## 3. Results

**Figure 1:**
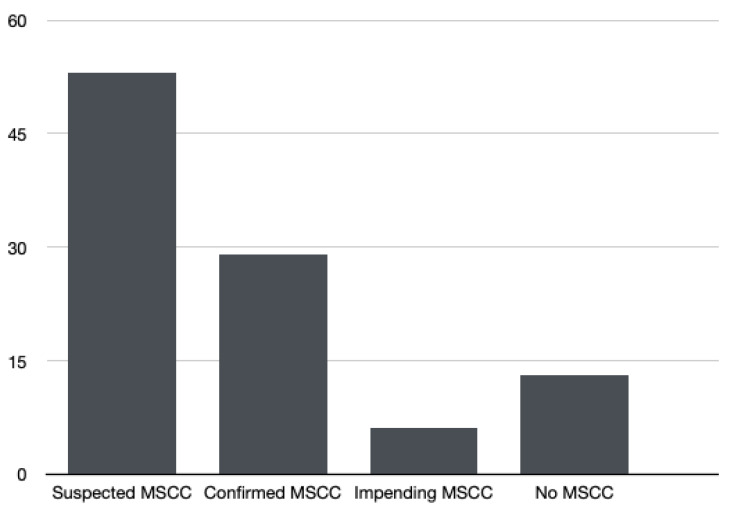
Distribution of the suspected malignant spinal cord compression (MSCC) cases.

In total, we identified 53 patients with suspected MSCC. Among them, 29 (54.7%) had confirmed MSCC in MRI imaging (Figure 1). Impending MSCC, defined as a clinical entity without neurological deficits in the presence of epidural tumour touching the spinal cord, was diagnosed in 6 patients (11.3%) [9].

The most common malignancies within the confirmed MSCC subset were lung (11 patients, 37.9%), breast (5 patients, 17.2%), and renal (3 patients, 10.3%), followed by prostate, myeloma, and carcinoma of unknown primary (2 patients (6.9%) each), as well as pancreatic, colorectal, lymphoma, and bladder (1 patient (3.4%) each) (Table 1).

Of our 53 patients entering the MSCC pathway, 44 patients (83%) presented to Accident and Emergency (A&E) initially, 7 (13%) to Ambulatory Care, 1 (2%) to Medway On Call Care (MedOCC), 1 (2%) to the Outpatient Day Unit. With varying areas of presentation, 22 patients (46%) were referred to the acute oncology service within 24 h. Only 21 (40%) patients were suspected of having MSCC in the first 24 h. This compares to 9 (17%) patients suspected between 24–72 h and 3 (6%) patients suspected after 72 h with the longest suspicion after six days. Seven patients (13%) were admitted after suspicion in an outpatient setting. Furthermore, 13 (24%) patients were never suspected of MSCC but had an MRI for other reasons, e.g., mechanical back pain, and subsequently entered the pathway. Out of all these patients, 13% were never suspected of MSCC but diagnosed on imaging. 

**Figure 2:**
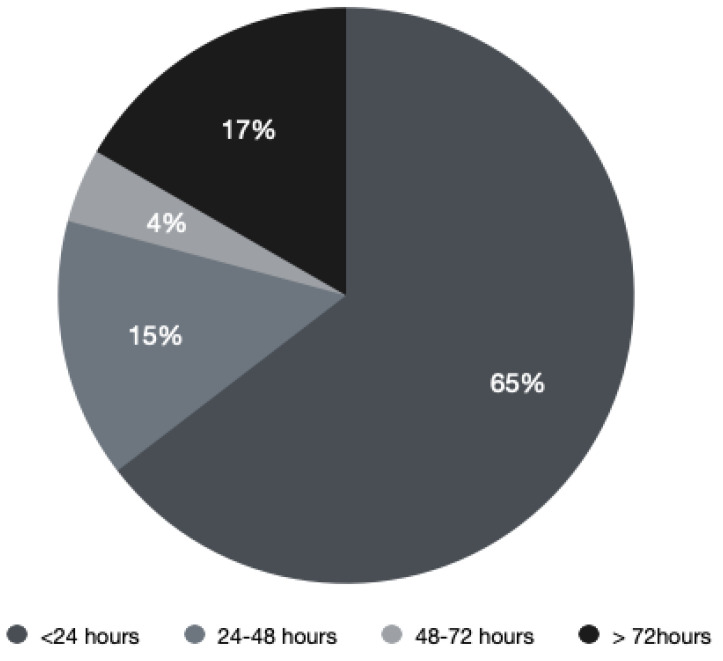
Time from admission to magnetic resonance imaging (MRI) in hours.

**Figure 3:**
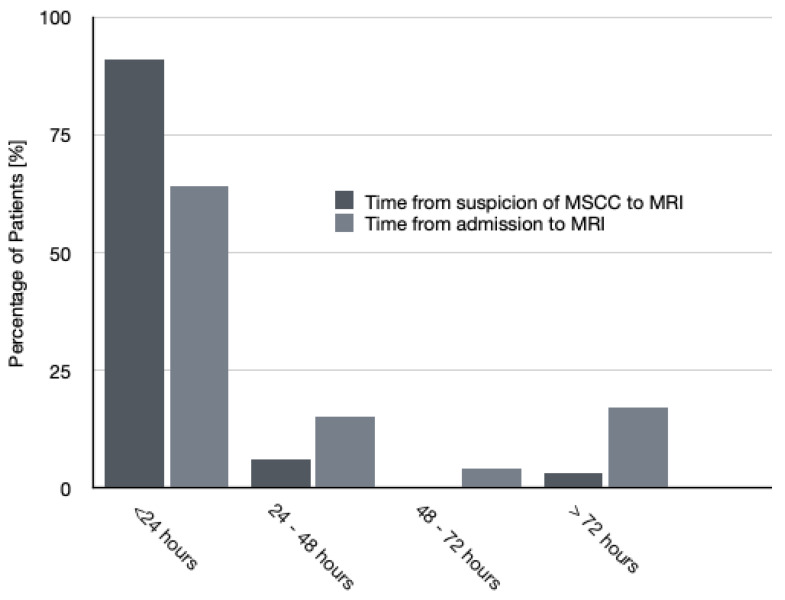
Comparison of time of magnetic resonance imaging (MRI) from admission and suspicion of malignant spinal cord compression (MSCC).

An MRI scan was performed in 48 patients (90.5%); the majority (31 patients, 64.6%) underwent the MRI within the first 24 h of admission, whereas 9 patients had the investigation between 24 and 72 h from the admission (Figure 2). In comparison, the time from suspicion of MSCC—rather than patient admission—to MRI is better with 32 patients (91%) receiving the MRI within 24 h. Figure 3 shows the comparison of the time of MRI from admission and suspicion of MSCC. Four patients (7.5%) had a Computerized Tomography (CT) scan instead of an MRI.

Following MRI, we assessed the timing of completion of the MRI report by a senior radiologist. This is demonstrated in Table 2. There were a range of timings from as little as 23 min to over 20 h. 

Twenty nine patients were diagnosed as confirmed MSCC. Among them, 6 (20.7%) were treated with surgical decompression, while 20 (69%) received RT and 3 (10.3%) best supportive care, respectively (Table 3).

Ten patients (36%) received definitive treatment within 24 h of diagnosis, 9 patients (32%) received treatment within 24–48 h of diagnosis, and 9 patients (32%) received treatment 72 h after diagnosis. Median time to surgery was 5 days (ranged between 2 and 8 days), whereas for RT 44.4 h (ranged between 24 and 72 h). Finally, all three patients that decided for symptom control were referred to the palliative care team within the first 24 h following the MRI scan. 

## 4. Discussion

Diagnosing and treating MSCC as an oncological emergency remains critical to preserving neurological function, quality of life, and survival for our patients. Our results demonstrated that multiple features of our clinical practice fulfilled the guidelines. However, there was an overall delay in recognizing and establishing a diagnosis of MSCC and involving relevant members of the oncology team, all of which led to a delay in definitive treatment. 

For earlier diagnosis, medical knowledge and understanding of the symptoms and signs of MSCC are essential. Back pain is commonly the first symptom of MSCC and occurs in up to 95% of patients; it can begin two to four months before progression of other neurological symptoms [1]. The pain, either localised or radicular, usually increases in severity over time and can be worsened on coughing or lying down due to increased pressure and distension of the epidural plexus. The NICE guideline advises that MSCC should be considered in patients with cancer that have severe unremitting or nocturnal pain in the cervical, thoracic, or lumbar spine. Box 1 summarises the NICE guideline on early symptoms and signs [4]. Unfortunately, literature suggests a lack of awareness of the pain in early stages of MSCC in primary and secondary care. This is due to cancer patients having a different significance and type of pain compared to pain in non-cancer patients—for example, their pain could be attributed to tumour progression.

Our study highlights this challenge of early diagnosis. Only 40% of our 53 patients entering the MSCC pathway were suspected of having MSCC in the first 24 h. Late suspicion of MSCC was highlighted by 6% suspected after 72 h with the longest suspected after six days. These results highlight that our institution is potentially missing an opportunity for early diagnosis, increasing the serious risk of loss of neurological function. This is further demonstrated by 13% of patients never suspected of MSCC but diagnosed on imaging. Though patients may present from a variety of referral pathways, 83% of this patient group initially presented via A&E. The majority of suspicion of MSCC in 24 h would be by doctors in this department, along with the acute medical team afterwards. Based on this, optimising education for doctors in these specialties would be vital to improving our speed in detecting MSCC within 24 h. Furthermore, it seems that there is a delay in referring to the acute oncology service, as only 46% of referrals were completed within the first 24 h of admission. Many patients are referred once an MRI confirms MSCC. However, 98% of patients are promptly reviewed by the acute oncology service within 24 h or on the next working day. The NICE guideline emphasizes referral to an MSCC coordinator within 24 h and we must improve our practice significantly [4]. Increased education of the management of MSCC and the role of acute oncology service in the A&E and acute medical teams may encourage earlier referral and a lower threshold for investigations leading to earlier diagnosis and treatment. Increased education for doctors on these issues is also well recommended in the literature [10,11].

Other symptoms of MSCC can be divided into motor, sensory, and autonomic deficits. Limb weakness can be the second most common symptom of MSCC as it affects 60–85% of patients [12]. Patients report a progressive change in their gait or weakness over days or weeks, which can be difficult to appreciate if regular clinical reviews are not undertaken. Furthermore, White et al. found that 50% of patients only presented when their mobility was affected, despite experiencing other symptoms for over two months [13]. This neurological change is considered an emergency for patients and doctors compared to other preceding symptoms, which further implicates diagnostic and treatment delay. Similarly, symptoms which can indicate a late consequence of MSCC are perineal anaesthesia in a saddle distribution and bladder and bowel dysfunction—this could be urinary retention, urinary or faecal incontinence, and constipation. Sensory symptoms are less common but patients may report paraesthesia extending up to 5 dermatomes below the level of compression [12]. A limitation of our data is less focus on symptoms experienced by patients and examinations performed by doctors to exclude MSCC. This includes neurological examination and patients’ ability to walk—which is often missed out in initial assessment of MSCC. This would be vital to confirm in order to further understand the diagnostic delays found in our results. It would also provide context for educational training given to doctors and areas for improvement.

Box 1 **The National Institute for Health and Care Excellence (NICE) guidelines on early symptoms and signs of malignant spinal cord compression (MSCC) [4].**

Contact the MSCC coordinator urgently (within 24 h) to discuss the care of patients with cancer and any of the following symptoms suggestive of spinal metastases:

1. Pain in the middle (thoracic) or upper (cervical) spine
2. Progressive lower (lumbar) spinal pain
3. Severe unremitting lower spinal pain
4. Spinal pain aggravated by straining (for example, at stool, or when coughing or sneezing)
5. Localised spinal tenderness
6. Nocturnal spinal pain preventing sleep

Contact the MSCC coordinator immediately to discuss the care of patients with cancer and symptoms suggestive of spinal metastases who have any of the following neurological symptoms or signs suggestive of MSCC, and view them as an oncological emergency:

1. Neurological symptoms including radicular pain, any limb weakness, difficulty in walking, sensory loss or bladder or bowel dysfunction
2. Neurological signs of spinal cord or cauda equina compression

Perform frequent clinical reviews of patients with cancer who develop lower spinal pain that is clinically thought to be of non-specific origin (that is, it is not progressive, severe or aggravated by straining and has no accompanying neurological symptoms). In particular, look for:

[1] Development of progressive pain or other symptoms suggestive of spinal metastases (contact the MSCC coordinator within 24 h), or
[2] Development of neurological symptoms or signs suggestive of MSCC (contact the MSCC coordinator immediately)

Perform frequent clinical reviews of patients without a prior diagnosis of cancer who develop suspicious spinal pain with or without neurological symptoms. Treat or refer patients with stable and mild symptoms by normal non-specific spinal pathways, or refer by cancer pathway if concerned. In particular, look for:

1. Development of progressive pain or other symptoms suggestive of spinal metastases (contact the MSCC coordinator within 24 h), or
2. Development of neurological symptoms or signs suggestive of MSCC (contact the MSCC coordinator immediately)

Timely access to MRI imaging will also improve diagnostic delays of MSCC. As per the NICE guideline, MRI remains the gold-standard to diagnosing MSCC with sensitivity of 93% and specificity of 97% and should be done within 24 h [4,12,14]. Unfortunately, literature shows that despite the non-invasive and highly effective investigation choice of MRI, many patients are still being diagnosed late with this. This differs from our study, where 91% of patients suspected of MSCC had their MRI within 24 h. However, this is reduced with 64% of patients receiving MRI within 24 h of admission. Consequently, the rapid availability of MRI imaging in our centre complies with the guidelines; however, there is a gap of clinical suspicion of MSCC when patients are admitted. This again links back to the delay in diagnosis of MSCC reported in the literature, which could be due to the history taking and clinical examination performed by doctors. Our objective should be to improve MRI imaging in MSCC patients so that it is within 24 h of admission.

Another issue highlighted by our data was timing of reporting of MRI as previously demonstrated in Table 2. There is no explicit time cut-off for this in the guidelines nor evidence in the literature; however, if both MRI and treatment need to be initiated within 24 h of suspicion, then delays in reporting will lead to delays in diagnosis. This can be further complicated if MRIs are reported out-of-hours and not picked up in time by the on-call team. Literature already demonstrates that there is a low percentage of patients diagnosed with MSCC along with its sub-optimal management on the weekends [1,15]. One of our patients had an MRI as an outpatient with a report only completed 72 h after. Though RT was delivered on the day the MRI was reported, there was still a delay which could be significant for symptom progression and consequently success of treatment. These delays could be due to the limited number of radiologists who are able to report MRIs in the hospital but this needs to be explored further. Therefore, to allow for treatment of MSCC as soon as possible, oncology centres should perhaps outline a clear pathway of when MRI should be performed and also reported by a senior radiologist. Furthermore, this pathway should include an MRI-imaging grading system of MSCC. Bilsky et al. developed a relevant 6-point grading system for MSCC/epidural spinal cord compression [16]. This provides a reliable and uniform reporting of MSCC, which can guide further treatment appropriately. A major limitation of our data is no inclusion of this grading system for MSCC diagnoses. Therefore, when collaborating with radiologists for a clear MSCC pathway, this system should be incorporated. 

**Figure 4:**
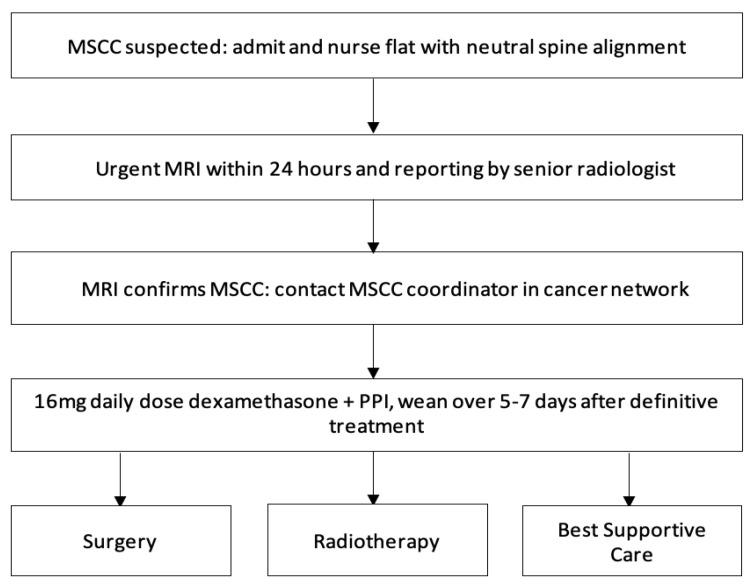
Diagnosis and management of malignant spinal cord compression (MSCC). Abbreviations: MSCC: Malignant spinal cord compression; PPIs: Proton Pump Inhibitors.

As well as early diagnosis, treatment of MSCC should be delivered within 24 h. The treatment is a combination of high-dose steroids, RT, surgical intervention, and extensive rehabilitation, and must be initiated within 24 h of diagnosis to prevent further neurological decline [4,7]. Our study shows significant improvement is needed with only 36% of patients receiving treatment less than 24 h after MRI diagnosis. Figure 4 summarizes the diagnosis and treatment pathway. While awaiting definitive management, such as RT and surgery, high doses of steroids provide analgesia, decrease spinal cord vasogenic oedema, and the secondary complication of reduced arterial flow and therefore, prevent further neurological deterioration [17]. In some cases, it can decompress the tumour causing the compression [7,18]. Steroids should be given immediately within 12 h of diagnosis for optimum efficacy and weaned after RT or surgery over 5–7 days to avoid side-effects [7]. Our study could be improved by inclusion of when steroids were administered, inclusion of Proton Pump Inhibitors (PPIs), and whether they were adequately weaned following definitive treatment.

Surgery is indicated in patients for surgical decompression and spinal stabilisation [4]. Surgical decompression followed by adjuvant RT has shown more favourable outcomes than RT alone in the literature. A randomised trial by Patchell et al. showed more patients were able to walk and for significantly longer when treated with surgery rather than RT alone [19]. Furthermore, a meta-analysis found similar results with surgery and adjuvant RT improving ambulation and pain relief along with a higher difference of 1-year survival of 41% compared to 24% with RT alone [20]. Patients should have a prognosis of more than six months to be considered for surgery [7]. However, surgery can lead to complications such as pulmonary embolism, infections including postoperative pneumonia, cerebrospinal fluid leaks, and major bleeding [7,21]. This may account for reluctance of neurosurgeons to operate and explain the findings in our study with 21% of patients receiving surgery at a tertiary care hospital.

Six (20.7%) patients underwent decompressive surgery and this was influenced by a higher severity of symptoms, better performance status, and higher life expectancy. Only 33% of these patients underwent decompressive surgery within 48 h of MRI diagnosis with the remaining patients being operated on between six and eight days. Delays were due to no availability of hospital beds in the tertiary care hospital. However, once transferred, 67% of patients were operated on within 24 h. This is a challenge for our two centres, as inter-hospital transfer is often difficult to control and can be very variable and unpredictable. Furthermore, neurosurgical referral is completed online and a limitation of our study is no data on timings of referrals done by the A&E and medical team. While the online referral is faster than verbally calling the neurosurgical department, not all doctors have login details or updates by the neurosurgical team are not checked on a regular basis. Literature shows earlier surgery within 24 h results in improved neurological outcomes and the NICE guideline also encourages this timing [4,22]. For surgery within 24 h, both centres should organise a neurosurgical pathway where these patients are given priority for transfer within 24 h and education for doctors on the whole diagnostic process, including creating the online referral and regularly checking for updates.

**Figure 5:**
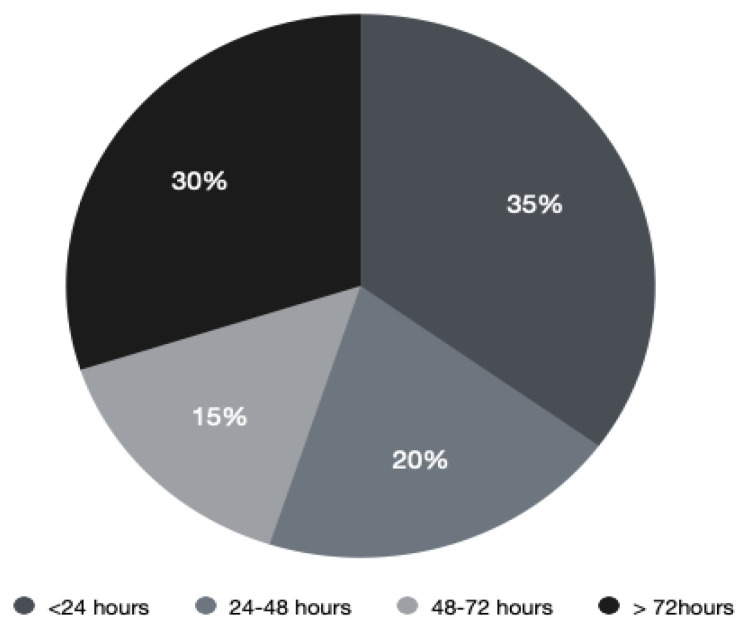
Time from confirmed malignant spinal cord compression (MSCC) to radiotherapy (RT).

RT is highly effective in MSCC by providing analgesia and preventing further neurological deterioration [12]. It is indicated within 24 h of diagnosis and can provide benefit to patients who are not surgical candidates [4,7,12]. Fractions of RT given depend on the primary malignancy and its systemic burden, duration of symptoms, and prognosis [4,7]. The majority of patients in our study (20 patients, 69%) received RT and this was also influenced by performance status and life expectancy of patients. However, only 35% were irradiated less than 24 h after MRI diagnosis. Figure 5 shows the time from confirmed MSCC to RT. There are numerous reasons for the delay in receiving RT. Firstly, RT is not offered by the Medway Maritime Hospital and so requires communication between two institutions in Kent (Medway Maritime Hospital and Maidstone and Tunbridge Wells NHS Trust). This is further complicated by the absence of a specific contact to direct referrals to. Therefore, referrals are directed to the clinical oncologist on-call, which can be difficult to organise over the phone in a timely manner. Challenging access to RT then affects the practical logistics—confirmation RT can be delivered, its availability due to demand and transporting patients on time. Another contributing factor to delay is awaiting neurosurgical outcomes on whether the patient is a candidate for surgery. As well as the online neurosurgical referral, patients are also discussed in a multi-disciplinary meeting (MDM) towards the end of the week to decide if there is spinal instability. For this reason, RT is often delayed until there is a final decision on surgery. 

It is clear that a MSCC referral pathway needs to be more streamlined for improved treatment outcomes. Table 4 summarises the treatment options and the delays from diagnosis to treatment. Clinical oncologists should be included as part of the neurosurgical pathway recommended above in order to improve communication between three different areas of expertise and treatment timing. It may be advisable to have an MSCC coordinator in the oncology centre along with representatives from both the clinical oncologist and neurosurgical teams to oversee the treatment pathway and improve clinical practice. This could be further implemented in an MDM for MSCC mid-week compared to later on. Having an MSCC coordinator could improve both the diagnostic and treatment pathways. In our study, delays in RT were also caused by MSCC confirmed out-of-hours or over the weekend, which led to late referrals to the acute oncology service, clinical oncologist and neurosurgical teams. An MSCC coordinator could provide teaching to junior doctors on the referral process and treatment pathway as part of their core teaching curriculum. Furthermore, referral forms can be created, which include clinical history, assessment, MRI report, and contact details of the neurosurgical and clinical oncologist teams to encourage A&E and medical specialties to refer to them all simultaneously. This form can then be emailed to the MSCC coordinator who can act as the primary point of referral to these specialties. A defined pathway such as this will improve access to definitive treatment and consequently improve neurological outcomes. 

**Figure 6:**
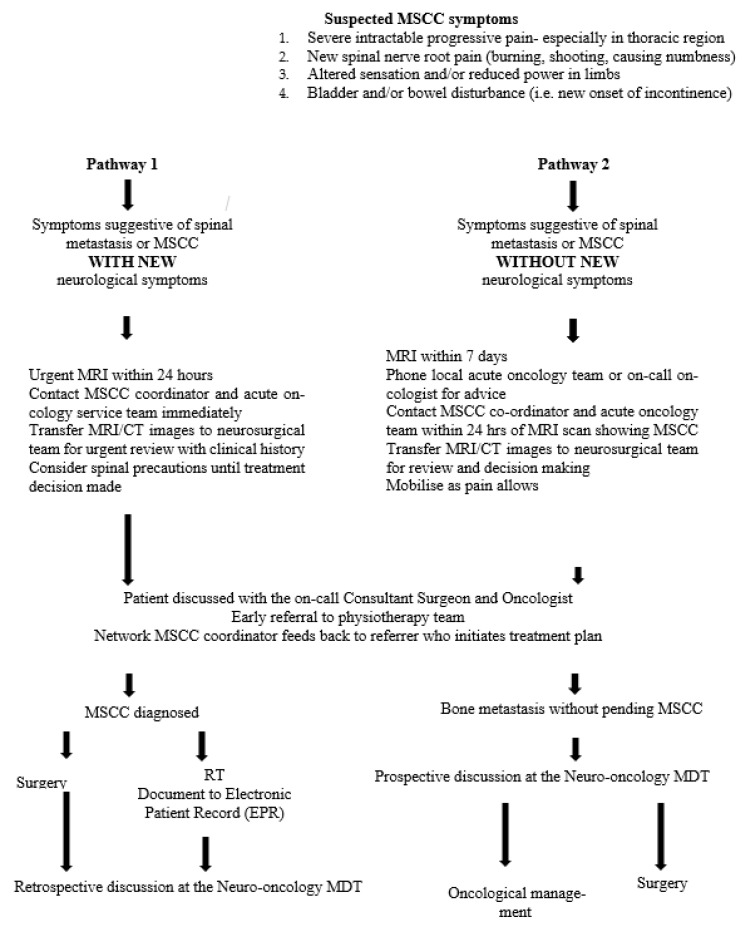
Medway Maritime Hospital updated malignant spinal cord compression (MSCC) guidelines. Abbreviations: MSCC: Malignant spinal cord compression; MDT: Multidisciplinary team; MRI: Magnetic resonance imaging; CT: Computerized tomography; RT: Radiotherapy.

Figure 6 summarises the updated local MSCC guidelines that we are planning to distribute based on our experience and the results of this audit.

## 5. Conclusions and Future Directions

MSCC represents an oncological emergency and clinicians should be aware of the potential long-term neurological impact. Urgent diagnosis and treatment is still challenging. MRI of the whole spine is the imaging method of choice that should be carried out within 24 h of clinical suspicion. Steroid therapy is administered immediately after the establishment of diagnosis, followed by definitive treatment, which may include any combination of surgery and/or RT. Treatment should ideally be initiated within 24 h of the confirmed MSCC. Our study demonstrates that MSCC is overall poorly understood amongst clinicians. It is evident that trainees require further teaching to improve their knowledge. Equally, oncological patients should be aware of the signs and symptoms of MSCC in order to optimise early detection.

In summary, formulation of a standard treatment protocol may be beneficial in assessing, auditing, and improving the standard of care in the acute management of patients presenting with MSCC. To aid this, we have developed an electronic MSCC form in the oncology *Electronic Patient Record* (EPR) system to document the management of MSCC more accurately. Furthermore, updated guidelines have been written to provide clearer guidance to the clinical teams seeing and assessing these patients when they first present in our hospital. To avoid diagnostic and therapeutic delays, early referral to the local acute oncology team to co-ordinate the patient pathway is critical. Overall, the gold standard pathway would include a dedicated team, including a coordinator, radiologist, clinical oncologist, and neurosurgeon to oversee the treatment pathway and improve clinical practice.

## Figures and Tables

**Table 1:** Cancer types of the patients with confirmed malignant spinal cord compression (MSCC).

Cancer Type	Number of Patients	% of Patients with Confirmed MSCC
Lung	11	37.9
Breast	5	17.2
Renal	3	10.3
Prostate	2	6.9
Myeloma	2	6.9
Carcinoma of unknown primary	2	6.9
Pancreatic	1	3.4
Colorectal	1	3.4
Lymphoma	1	3.4
Bladder	1	3.4

**Table 2:** Time from the performed magnetic resonance imaging (MRI) to the radiology report.

Hours	Number of Scans Reported
<1	2
1–2	16
2–3	14
3–4	8
4–5	2
5–10	3
10–20	3

**Table 3:** Treatment of the patients with confirmed malignant spinal cord compression (MSCC).

Type of MSCC	Number of Cases	Treatment		
		Surgery	Radiotherapy	Best Supportive Care
Confirmed	29	6 (20.7%)	20 (69%)	3 (10.3%)
Impending	6	1 (16.7%)	4 (66.6%)	1 (16.7%)
Total	35	7 (20%)	24 (68.6%)	4 (11.4%)

**Table 4:** Time from MRI to treatment *.

Surgery		RT			Best Supportive Care	
<48 h	>48 h	<24 h	24–48 h	>72 h	<24 h	>24 h
2 (40%)	3 (60%)	7 (35%)	7 (35%)	6 (30%)	3 (100%)	0

* One surgical patient was not included, as data not available at time of report.

## Data Availability

Data supporting reported results can be found in the electronic patient report (EPR) of Medway Maritime Hospital.
